# Effects of liposomal-curcumin on five opportunistic bacterial strains found in the equine hindgut - preliminary study

**DOI:** 10.1186/s40781-017-0138-4

**Published:** 2017-06-12

**Authors:** S. D. Bland, E. B. Venable, J. L. McPherson, R. L. Atkinson

**Affiliations:** Department of Animal Science, Food & Nutrition, Southern Illinois University, Carbondale, IL 62901 USA

**Keywords:** *Clostridium*, *Escherichia coli*, Equine, Microbiota, Nutraceutical, *Streptococcus*

## Abstract

**Background:**

The horse intestinal tract is sensitive and contains a highly complex microbial population. A shift in the microbial population can lead to various issues such as inflammation and colic. The use of nutraceuticals in the equine industry is on the rise and curcumin is thought to possess antimicrobial properties that may help to minimize the proliferation of opportunistic bacteria.

**Methods:**

Four cecally-cannulated horses were utilized to determine the optimal dose of liposomal-curcumin (LIPC) on reducing *Streptococcus bovis/equinus* complex (SBEC), *Escherichia coli* K-12, *Escherichia coli* general, *Clostridium difficile*, and *Clostridium perfringens* in the equine hindgut without adversely affecting cecal characteristics. In the first study cecal fluid was collected from each horse and composited for an in vitro*,* 24 h batch culture to examine LIPC at four different dosages (15, 20, 25, and 30 g) in a completely randomized design. A subsequent in vivo 4 × 4 Latin square design study was conducted to evaluate no LIPC (control, CON) or LIPC dosed at 15, 25, and 35 g per day (dosages determined from in vitro results) for 9 days on the efficacy of LIPC on selected bacterial strains, pH, and volatile fatty acids. Each period was 14 days with 9 d for acclimation and 5 d withdrawal period.

**Results:**

In the in vitro study dosage had no effect (*P* ≥ 0.42) on *Clostridium* strains, but as the dose increased SBEC concentrations increased (*P* = 0.001). Concentrations of the *E. coli* strain varied with dose. In vivo, LIPC’s antimicrobial properties, at 15 g, significantly decreased (*P* = 0.02) SBEC when compared to 25 and 35 g dosages. *C. perfringens* decreased linearly (*P* = 0.03) as LIPC dose increased. Butyrate decreased linearly (*P* = 0.01) as LIPC dose increased.

**Conclusion:**

Further studies should be conducted with a longer dosing period to examine the antimicrobial properties of curcumin without adversely affecting cecal characteristics.

## Background

Horses may suffer from inflammation in their gastrointestinal (GI) tract, such as colic, enterocolitis, diarrhea, and inflammatory bowel disease [1]. It has been reported that seasonal environmental changes, viruses, intestinal parasites, and infectious agents, such as *C. difficile* and *C. perfringens*, are a few causes of colitis, or inflammation of the large intestine, and diarrhea [2–4]. The intestinal tract contains a highly complex, yet sensitive microbial population, and a shift in this population can lead to serious gastrointestinal issues for horses [2]. *C. perfringens, C. difficile, E. coli* general and K-12*,* and *Streptococcus bovis/equinus* complex (SBEC) are common opportunistic bacteria found in the hindgut. In a study examining the hindgut of horses with starch-induced laminitis, it has been suggested that SBEC could precede the onset of laminitis due to the increased concentrations of *Streptococcus spp*. isolates [5].

Oral administration of nonselective nonsteroidal anti-inflammatory drugs (NSAIDs) to horses, with gastrointestinal disease, is a common practice in veterinary medicine [6]. However, studies examining the effects of NSAIDs suggest that these drugs are associated with adverse gastrointestinal effects [6]. Additionally, studies have shown that NSAIDs, in vitro, can affect hindgut mobility, which could lead to a change in the pH, volatile fatty acid concentration, and overall digestion [7]. Since nonselective NSAIDs are more preferable in equine medicine [6], the potential of additional adverse effects could lead to dysbiosis of the hindgut microbiota. The utilization of nutraceuticals, in an effort to prevent the aforementioned dysbiosis, offer a potential therapy to mitigate the adverse side effects associated with the traditional therapeutic use of NSAIDs.

Curcumin is the active ingredient in turmeric [*Curcuma longa*], and in human [8], chicken [9], and horse [10] studies it has demonstrated antimicrobial properties. While the targeted nature of the antimicrobial effects are yet unknown in horses; curcumin’s antimicrobial properties may help to minimize the proliferation of opportunistic bacteria in the equine hindgut. However, while curcumin could potentially be an alternative treatment for a wide variety of diseases, it has poor bioavailability [11]. Observed in humans [8], and mice [12], curcumin’s poor bioavailability is due to its hydrophobic properties and quick elimination from the body [12]. However, studies have speculated that encapsulating curcumin in liposomes could increase its bioavailability [12]. The objective of this research was to evaluate the antimicrobial properties of liposomal-curcumin and its effect on cecal characteristics.

## Methods

Four cecally-cannulated [13] horses, one gelding and three mares, weighing 522.95 ± 16.59 kg and having a body condition score (BCS) of 5.5 ± 0.5 on a scale of 1-9, with nine being obese, were used for the in vitro batch culture experiment and in the in vivo study. Southern Illinois University Animal Care and Use Committee (Protocol 14-048) approved care and handling of animals used in this study.

### In vitro

The in vitro 24 h batch culture examined the effect of dose on bacteria concentrations when supplementing liposomal-curcumin (LIPC). Sixteen 125 mL Erhlenmeyer flasks were randomly assigned one of the following treatments in quadruplicate: 1) LIPC at the recommended dose of 15 g, (15); 2) 20 g of LIPC, (20); 3) 25 g of LIPC (25); or 4) 30 g of LIPC, (30). Based on the recommended dosage of 500 mg/g of turmeric at 15 g per 454.54 kg horse [10], the selected treatments were increased by 5 g, up to twice the recommended dose.

Composited cecal fluid mixed with McDougall’s buffer, at a 1:4 ratio [12], was poured (50 mL) into 16 separate 125 mL Erlenmeyer flasks, degassed with carbon dioxide (CO_2_), and placed in a water bath at 39 °C. The 125 mL Erlenmeyer flasks also contained 0.50 g [14] of ground alfalfa hay. Flasks were manually shaken every 2 h for 24 h.

Samples were collected at 0 and 24 h, pH measured (Oakton pH 110 Advanced Portable Meter (Vernon Hills, IL), stored in a 15 mL conical tube, and frozen at −80 °C for later analysis, including deoxyribonucleic acid (DNA) extraction (PowerSoil Mo Bio DNA Extraction Kits (Mo Bio Laboratories, Carlsbad, CA) and qPCR (Bio-Rad MyiQ Optical System Software 2.0). A Nano Drop ND-1000 Spectrophotometer (Wilmington, DW) assessed all DNA extractions for concentration and quality prior to PCR.

Performed in triplicates, all real-time PCR runs and each reaction mixture was prepared using Maxima SYBR Green/ROX qPCR (Thermo Scientific, Waltham, MA). The five opportunistic bacteria, *E. coli* general and K-12 [15] *C. difficile* [16, 17], *C. perfringens* [18], and SBEC [19] were amplified using a CFX96 Touch Real-Time PCR Detection System (Bio-Rad Laboratories, Inc., Hercules, CA), quantity analysis was performed by calculating the absolute value, using the cycle threshold. The primers sequences used are in Table 1.

**Table 1:** Forward and Reverse Primers used for real time PCR

Strains	Forward Primers (5′ – 3′)	Reverse Primers (5′ – 3′)
SBEC	GCCTACATGAAGTCGGAATCG	TACAAGGCCCGGGAACGTA
*C. difficile*	CAAGTTGAGCGATTTACTTCGGTAA	CTAATCAGACGCGGGTCCAT
*C. perfringens*	AAATGTAACAGCAGGGGCA	TGAAATTGCAGCAACTCTAGC
*E. coli, general*	GTTAATACCTTTGCTCATTGA	ACCAGGGTATCTAATCCTGTT
*E. coli K12*	GCTACAATGGCGCATACAAA	TTCATGGAGTCGAGTTGCAG

### In vivo

Four cecally-cannulated horses were utilized in a 4 × 4 Latin square to evaluate increasing doses of LIPC on the concentrations of the same opportunistic bacteria stated in the in vitro and to examine cecal characteristics, such as volatile fatty acids (VFA) and ammonia nitrate (NH_3_) concentrations. One of four treatments: 1) no LIPC, (0); 2) 15 g LIPC, recommended dose, (15); 3) 25 g LIPC (25); or 4) 35 g LIPC (35) for a total of 7500 mg, 12,500 mg, and 17,500 mg, respectively, of the active ingredient dosed daily, were randomly assigned to horses. Horses were fed 0.90-1.36 kg of Strategy® (Purina Mills, St. Louis, MO), once daily at 0600 and the treatments were top-dressed on the grain, for delivery and to maintain a BCS of 5-6. Post grain and treatment consumption, horses were then turned out to pasture (predominantly K31 Tall Fescue) and allowed to graze until 1600. This was the daily procedure with the exception for d 9 of each period, during which they were stalled all day and had *ab libitum* access to hay and water after complete consumption of Strategy® and treatment.

Each period was 14 days with a 9 d acclimation period and a 5 d withdrawal period [10, 20]. Cecal fluid was collected at 0 h on d 0 and 8, and again on d 9 at 0, 3, 6, 9, 12, 15, 18, and 21 h. Whole cecal contents (100 mL) were collected, pH recorded (Oakton pH 110 Advanced Portable Meter (Vernon Hills, IL), subsampled (15 mL), and immediately frozen for later analysis of opportunistic bacteria. On d nine, after pH was recorded, contents were filtered through eight layers of cheesecloth into a 15 mL collection tube and immediately frozen for later analysis of VFA and ammonia concentrations. Cecal NH_3_ concentrations were determined by the phenol-hypochlorite procedure [21]. VFA concentrations were determined [22] using a Shimadzu GC-2010 gas chromatograph (Shimadzu Scientific Instruments, Inc., Columbia, MD) and internal standards made with 2-ethyl butyrate [22].

Blood was also collected via jugular venipuncture on d 0 and 8 into a vacutainer serum separator tube and a 7.5% Ethylenediaminetetraacetic acid (EDTA) tube (Coviden, Mansfield, MA) for chemistry panel analysis, and complete blood count analysis, respectively.

### Statistical analysis

The bacterial concentrations from in vitro experiment were analyzed as a completely randomized design using the MIXED procedure of SAS (SAS 9.4 Inst., Inc., Cary, NC).

For the in vivo experiment, bacterial concentrations, chemistry panel data, and complete blood count data were analyzed using the MIXED procedure of SAS (SAS 9.4 Inst., Inc., Cary, NC) using the model for a Latin square design with a Tukey *post-hoc* adjustment. Cecal fermentation data (NH_3_, pH, and VFA) were analyzed using the MIXED procedure of SAS for repeated measures. An autoregressive covariance structure (AR1 of the MIXED procedure of SAS) was determined to be most appropriate based on Akaike’s Information Criterion. Comparisons of main effects were determined using least square means and Fisher’s protected LSD. Calculation of coefficients for linear orthogonal polynomials with unequal spacing was done using IML of SAS [23]. Significance was set at (*P* ≤ 0.05) and tendency was set at (*P* ≤ 0.10).

## Results

### In vitro

The bacteria concentrations for the in vitro study are summarized in Table 2. Every flask had a pH within the normal equine cecum pH range of 6.5-7.1 [24]. Concentrations of SBEC were significantly lower (*P* < 0.0001) at the recommended dose (15) when compared to the 20, 25, and 30 dose treatments. *E. coli* substrain K-12 concentrations increased (*P* = 0.01) in the 25 and 30 treatments compared to 15 and 20 treatments. Concentrations of *E. coli* general were significantly less (*P* = 0.03) for 15, 20, and 30 compared to the 25 treatment.

**Table 2:** The effects of liposomal-curcumin on bacteria (ng/μL) found in equine cecal fluid, in vitro (24 h)^a^

	Treatment^1^					
Strains	15	20	25	30	SEM	*P*-value
SBEC	5.49E + 09^a^	1.79E + 11^b^	5.07E + 13^c^	2.60E + 12^d^	2.73E + 07	0.0001
*E. coli* K-12	7.93E + 03^a^	1.30E + 04^a^	2.86E + 06^b^	3.39E + 06^b^	7.67E + 05	0.01
*E. coli* general	1.30E + 02^a^	9.60E + 01^a^	2.08E + 04^b^	4.81E + 03^a^	4.85E + 01	0.03
*C. difficile*	2.14E + 03	1.74E + 03	2.15E + 01	1.07	1.33E + 03	0.56
*C. perfringens*	5.20E-01	1.74E-02	2.06E-01	6.56E + 01	3.20E + 01	0.42

^a^Data are means of 4 jars per replicate. ^a-d^Means within a row with different superscripts differ significantly (*P* < 0.05)

^1^Treatments: 15 g; 20 g; 25 g; 30 g of 95% liposomal-curcumin

### In vivo

Based on the results of the batch culture, the authors decided to investigate 15 g, 25 g, and 35 g of 95% LIPC. SBEC bacterial concentrations increased linearly (*P* = 0.008) as LIPC dose increased (Table 3). However, as the dose of LIPC increased, the concentration of *C. perfringens* decreased linearly (*P* = 0.03).

**Table 3:** Effects of liposomal-curcumin on opportunistic bacteria (ng/uL) found in equine cecal fluid and on cecal fluid characteristics (9 d)^a^

	Treatment^1^					*P* -value	
	0	15	25	35	SEM	TRT^2^	LIN^3^
Bacterial strains							
SBEC	13.00^ab^	12.73^a^	13.68^bc^	14.12^c^	0.25	0.02	0.008
*E. coli* K-12	20.46	19.16	19.73	20.50	1.49	0.20	0.96
*E. coli* general	32.64	32.77	32.21	33.27	0.37	0.94	0.79
*C. difficile*	29.61	29.68	30.63	31.15	1.01	0.62	0.25
*C. perfringens*	49.18	46.36	45.97	43.51	1.36	0.12	0.03
Cecal characteristics							
pH	6.71	6.68	6.68	6.67	0.03	0.82	0.38
Ammonia, mg/dL	15.89	15.6	9.94	12.25	2.12	0.21	0.11
Total VFA, mM	51.59	71.15	73.68	65.32	6.14	0.11	0.10
VFA, mol/100 mol							
Acetate	35.53	36.64	36.64	40.91	1.98	0.28	0.10
Propionate	49.68	52.75	54.75	50.15	1.96	0.34	0.62
Isobutyrate	3.97	1.67	1.31	0.59	1.90	0.54	0.19
Butyrate	10.63	9.57	8.09	8.46	0.62	0.06	0.01
Isovalerate	0.40	0.13	0.14	0.08	0.09	0.10	0.03
Valerate	0.68^a^	0.32^b^	0.28^b^	0.24^b^	0.09	0.02	0.005

^a^Data are means of 4 cannulated horses per replicate. ^a-c^Means within a row with different superscripts differ significantly (*P* < 0.05)

^1^Treatments: 0 = control (no nutraceutical); 15 = 15 g; 25 = 25 g; 35 = 35 g of 500 mg/g 95% liposomal-curcumin

^2^ *P-*value for treatment means

^3^ *P-*value for linear contrast

Cecal fluid pH and ammonia concentration were not significant among treatments (*P* = 0.82) and (*P* = 0.21), respectively (Table 3). However, ammonia concentrations decreased numerically in a linear fashion as LIPC dose increased. Valerate was significantly different (*P* = 0.02) among treatments with 0 having the greatest concentration compared to all other treatments. Moreover, valerate decreased linearly (*P* = 0.005) as LIPC dose increased. As LIPC dose increased, butyrate and iso-valerate decreased linearly (*P ≤* 0.03). However, acetate tended to increase linearly (*P* < 0.10), as the dose of LIPC increased. Lastly, increasing doses of LIPC tended (*P* = 0.10) to linearly increase total VFA concentrations when compared to 0.

## Discussion

### In vitro

Previous work, with human subjects, showed *E. coli* substrain K-12 possesses curcumin-converting activity, allowing this substrain to utilize curcumin as a substrate for growth [8]. It is possible that in the current study, increasing the dosage of LIPC increased the concentration of curcumin that *E. coli* general and K-12 could utilize as a substrate thus, allowing for an increase in these bacterial concentrations.

### In vivo

Although increasing the dose of LIPC decreased *C. perfringens*, the observation that increasing the dose also increases SBEC and *C. difficile* would suggest that there may be no additional benefit of dosing LIPC above the recommended rate and could lead to potential problems, such as an dysbiosis of the hindgut microbiota leading to colic, diarrhea, and enterocolitis. In addition, this would also suggest that the nutraceutical is not compromised in the stomach or small intestine during the digestion process before reaching the cecum.

The tendencies to increase acetate and total VFAs would suggest that a longer dosing period of LIPC might increase fiber digestibility [25]; however, a decrease in butyrate may decrease intestinal lining repair. In addition, when dosing higher than the recommended dose (15 g) for long periods, caretakers should take caution. The numerical decrease in ammonia and isobuytrate along with a decrease in isovalerate suggests that rate of protein degradation may decrease when LIPC is administered above the recommended dose for longer periods.

## Conclusion

The utilization of the nutraceutical, liposomal-curcumin, in an effort to prevent microbial dysbiosis, was thought to offer a potential therapy to mitigate the adverse side effects associated with the traditional therapeutic use of NSAIDs. However, the results of this study would suggest that liposomal-curcumin at doses above the recommended rate have the potential to increase the concentration of opportunistic bacteria, which would contribute to microbial dysbiosis rather than mitigate it. These preliminary data provide some insight of the effects of liposomal-curcumin on selected opportunistic bacteria. A more comprehensive and thorough examination of the cecal microbiota is needed understand the antimicrobial effects of the active ingredient in liposomal-curcumin on the equine microbiota. Additionally, further research is needed to assess long-term effects of the active ingredient in liposomal-curcumin on digestion because of the decrease in butyrate production.

## Abbreviations

BCS: Body condition score
CO_2_: Carbon dioxide
CON: Control
DNA: Deoxyribonucleic acid
EDTA: Ethylenediaminetetraacetic acid
GI: Gastrointestinal
LIPC: Liposomal-curcumin
NH_3_: Ammonia nitrate
NSAIDs: Nonselective nonsteroidal anti-inflammatory drugs
SBEC: *Streptococcus bovis/equinus* complex
VFA: Volatile fatty acids
